# Multi-channel volume density neural radiance field for hyperspectral imaging

**DOI:** 10.1038/s41598-025-00877-8

**Published:** 2025-05-09

**Authors:** Runchuan Ma, Sailing He

**Affiliations:** 1 National Engineering Research Center for Optical Instruments, Centre for Optical and Electromagnetic Research, College of Optical Science and Engineering, Zhejiang University, Hangzhou, 310058 China; 2 Department of Electromagnetic Engineering, School of Electrical Engineering, KTH Royal Institute of Technology, Stockholm, SE-100 44 Sweden

**Keywords:** 3D, Hyperspectral image, NeRF, Novel view synthesis, Optics and photonics, Computer science

## Abstract

Hyperspectral imaging and Neural Radiance Field (NeRF) can be combined in powerful ways. With limited hyperspectral images, NeRF can generate images of objects with spectral information from arbitrary viewpoints, which can effectively mitigate defects such as long acquisition time and difficulty in obtaining hyperspectral images. This paper addresses challenges in the application of NeRF methods in the hyperspectral domain, including local errors in convergence caused by noise. Leveraging the characteristics of hyperspectral data, we propose a neural radiance field method employing a multi-channel volume density distribution function. This approach alleviates issues during the generation of neural radiance fields from hyperspectral data, enhancing the robustness of hyperspectral neural radiance field methods across various scenarios, which can help downstream tasks such as discriminating objects more effectively than RGB methods. Experiments demonstrate that the proposed method generates superior hyperspectral images under diverse conditions, with a maximum PSNR 37.66 and a maximum SSIM 0.982.

## Introduction

Hyperspectral data is renowned for its abundance of spectral channel information and high spectral resolution. Since its inception, hyperspectral imagery (see e.g.^1–4^) has found widespread applications in various fields, including combining with artificial intelligence^5,6^, remote sensing^7,8^ and detection^9,10^. In contrast to images captured by common RGB cameras, hyperspectral data carries information beyond what RGB images can represent. It can discern phenomena when objects with same color in RGB images have different spectra.

With the continuous advancement of 3D technology and computer performance, methods like image-based 3D reconstruction have been increasingly applied across diverse domains. In this domain, Structure from Motion (SfM)^11^ is a crucial research direction. This method extracts feature points from images taken from different perspectives, matches them, and uses epipolar geometry to calculate the positions of these points, thereby accomplishing 3D reconstruction. Challenges in such methods include the sparsity of feature points and ineffective handling of texture-deficient regions. In recent years, the emerging Neural Radiance Field^12^ technology has gained substantial attention as a method for synthesizing new perspectives based on multiple-view images. This technique effectively addresses texture-deficient regions and produces high-quality new perspective images, overcoming the limitations of traditional methods. The development of image-based 3D technologies has driven progress in various fields like pose estimation^13–15^, 3D modeling^14,16,17^, and AR&VR^16^ (Augmented Reality and Virtual Reality).

The integration of the neural radiance field method with hyperspectral imagery holds substantial application prospects and research value. One of the most important advantages of hyperspectral data against RGB data is that hyperspectral images can easily discriminate some features which are similar in RGB. For object recognition tasks on special objects under complicated scenes, the RGB features of the target may not be conspicuous enough for deep learning model to train and recognize, usually causing low recall. This problem can be alleviated by using hyperspectral images. Moreover, models trained on 2D hyperspectral images have difficulty handling partly overlapping objects with same spectrum features in different distance, and thus 3D features are needed. Material recognition tasks can also benefit from hyperspectral data of 3D features, for a clearer boundary of a specific material can be shown in 3D, alleviating the problem caused by overlapping or mixing.

Earlier experiments^18–21^ and previously proposed dataset enhancement methods based on Neural Radiance Field (NeRF)^12^ technology have successfully introduced hyperspectral data into the NeRF domain, yielding positive outcomes. Due to the significantly higher information contained by hyperspectral images compared to RGB images and the susceptibility to noise and inconsistencies during the capture process, NeRF sometimes struggles to effectively reconstruct the three-dimensional features of hyperspectral data. This is manifested as noise in the form of haze, points, stripes, etc., which adversely affects the quality of synthesized perspectives.

This paper addresses these issues by proposing a method employing a multi-channel volume density distribution function for rendering. This method enhances the model’s representational capability and flexibility. By suppressing the interference of noise and inconsistencies with the model’s three-dimensional representation, the proposed method more effectively utilizes the abundant three-dimensional feature information in hyperspectral images, thereby improving NeRF’s reconstruction performance in the hyperspectral domain.

Through experimentation, this paper empirically demonstrates that the proposed method consistently outperforms the original neural radiance field technology in generating hyperspectral images under diverse conditions.

Main contributions

- We proposed a new rendering method for hyperspectral data using neural radiance fields, specifically the multi-channel volume density distribution function rendering method.
- We validated that this method enhances performance to some extent across various network parameters and training set sizes without requiring a significant increase in parameters.
- We explored the generation effects of this method for different spectral channel numbers. The results indicate a significant improvement in performance when dealing with multiple channels compared to fewer channels, demonstrating the effectiveness of this method for hyperspectral datasets.

### Related work

#### Hyperspectral image research

Hyperspectral images have been widely applied in various fields, including remote sensing and artificial intelligence. In the remote sensing domain, hyperspectral images are highly valued and play a crucial role in different detection tasks^7,8^. In the field of artificial intelligence, hyperspectral images find applications in various areas, as discussed in the following. Hyperspectral images can be acquired by cameras with filters^22^, which scan each channel of the spectrum and concatenate them together. Hyperspectral imaging by scanning takes minutes to take one image, which is much slower then RGB cameras. An advanced approach is compressive imaging^23^, the camera takes a snapshot and the hyperspectral images are recovered by algorithms afterward. Although snapshot hyperspectral imaging achieves a great success on imaging speed, recovering the hyperspectral image from the snapshot still takes a significant amount of time, while the quality of recovered images is still lower than scanning hyperspectral cameras. Another related field is active 3D hyperspectral imaging. This method requires the use of additional equipment to project onto the target, enabling the hyperspectral camera to acquire three-dimensional information, as discussed by J.Luo et al.^24^. While this method allows end-to-end acquisition of three-dimensional information, it has higher equipment requirements and usage conditions compared to fully image-based methods.

Another important area is hyperspectral image processing such as anomaly detection.There are a lot of related works based on traditional methods^25,26^ and achieved excellent results. R.Zhao et al.^27^ first proposed an anomaly detection algorithm without background feature extraction. R.Zhao et al.^28^ proposed a robust background regression based score estimation algorithm for hyperspectral anomaly detection, showing robust results. They also proposed another hyperspectral anomaly detection via a sparsity score estimation framework^29^, the experimental results show that the proposed framework achieves a superior performance compared to some of the state-of-the-art anomaly detection methods. There are also methods based on deep learning techniques, such as in the work^30^ by R.Zhao et al. they utilized fully convolutional auto encode neural network and achieved superior performance. Deep learning method can also be used to conduct tasks like image fusion and pansharpening^31^, in which an interesting pansharpening method was proposed.

#### 3D research based on images

Research on image-based 3D reconstruction consists mainly of two parts. One part involves more traditional feature-based 3D reconstruction methods, such as Structure from Motion (SfM)^11^. SfM generates the 3D information of objects from different perspectives, commonly using key point identification and matching for 3D reconstruction. Traditional key point identification methods like LIFT^32^, SIFT^33^, etc., have been tested for many years. With the increasing application of deep learning methods, key point identification based on deep learning has gained attention in recent years, with notable contributions from methods like SuperPoint^34^ and HSSPN^35^. However, methods based on key point identification and matching can only generate sparse results based on point clouds and have a poor performance on visualization. To address these problems, NeRF (Neural Radiance Fields) was proposed.

NeRF^12^ (Neural Radiance Fields) is an emerging neural network-based 3D perspective synthesis method. It can generate high-quality new perspective images using only images from different viewpoints for training. By training on multiple viewpoint images, the neural network can internally construct an implicit 3D representation, establishing the three-dimensional volume density distribution characteristics of objects at each spatial point. Rendering pixel by pixel using this information, NeRF can generate images from arbitrary angles. This method surpasses traditional methods like Structure from Motion (SfM).

Since NeRF was proposed, papers about NeRF have been publish to enhance this method in various ways. Mip-nerf^36^ effectively alleviated the problem of bluring and aliasing caused by different resolution, by efficiently rendering anti-aliased conical frustums instead of rays. By introducing a time variable, NeRF can now generate images for dynamic scenes, expanding its application from static to dynamic models^37,38^. D-NeRF^37^, a method that extends neural radiance fields to a dynamic domain, allowing to reconstruct and render novel images of objects under rigid and non-rigid motions from a single camera moving around the scene. Park K et al.^38^ present the first method capable of photorealistically reconstructing deformable scenes using photos/videos captured casually from mobile phones, augments NeRF by optimizing an additional continuous volumetric deformation field that warps each observed point into a canonical 5D NeRF. NeRF in the wild^39^ allows 3D reconstruction in real world outdoor scenes by solving the problem of inconsistent lighting and coverage problems. Some efforts focus on improving NeRF’s training or inference speed^40–42^, moving it from computationally intensive, time-consuming backend calculations toward real-time computation. Additionally, there are successful endeavors to enhance NeRF’s imaging resolution^43^, improving its ability to represent details in images and contributing to the generation of higher-quality images. Various directions, such as 3D modeling using NeRF^44^, extend its application beyond the realm of new perspective synthesis.

#### NeRF based on hyperspectral images

We study the integration of hyperspectral imaging with the Neural Radiance Fields (NeRF) method. A previous study^18^ successfully combined information from multiple cameras to achieve NeRF new perspective synthesis incorporating RGB, hyperspectral, infrared cameras, among others, with satisfactory results. However, the limitation of this method lies in its exclusive focus on forward 3D reconstruction experiments, lacking 360-degree multi-angle reconstruction.

Furthermore, R. Ma et al.^45^ explores the augmentation of limited hyperspectral datasets using NeRF to assist in 3D reconstruction. Conducting NeRF new perspective synthesis on multi-channel, high-resolution hyperspectral data, the article demonstrates the method’s effectiveness in augmenting datasets for improved 3D reconstruction.

The aim of this paper is to build upon previous work, enhancing the performance of NeRF in synthesizing new perspectives using hyperspectral datasets, particularly addressing challenges like noise and inconsistency inherent in hyperspectral data.

## Results

### Experimental design

The performance of our method and the original NeRF method on hyperspectral datasets was compared under various conditions. The images were captured in advance^35^. The basic configuration of our method inherits from NeRF, the activation function is chosen as Relu, and the optimizer is adam.

Initially, experiments were conducted on the effectiveness of using the multi-channel volume density distribution function $\sigma$ on hyperspectral datasets. The experiments were extended to datasets of different sizes, and comparisons were made with the original hyperspectral NeRF. Subsequently, we examined the impact of using our method on the reconstruction results of hyperspectral images with varying numbers of network parameters (referring here to the width of the fully connected network in the NeRF core). The goal of this experiment was to demonstrate that the advantages of our proposed method on hyperspectral datasets do not solely arise from the impact of simply increasing parameters.

Finally, the experiments explored the differences in the results between our method and the original hyperspectral NeRF when using different numbers of channels in hyperspectral images. These differences confirmed the correctness of our theoretical analysis.

The dataset used in this study is hyperspectral image dataset, comprising a total of 48 distinct perspectives. For the experiments in this paper, it will be divided into training and testing sets. Each perspective consists of images with 34 channels, and the central wavelength of each channel is distributed between 420nm and 750nm, with a spectral resolution of 10nm. For each channel, the image has 640 by 480 pixels.

Hyperspectral images were collected using an electro-optic tunable filter hyperspectral imaging system^35^, the hyperspectral camera is fixed on a tripod, and the turntable is controlled by a control software to rotate.

### Experiment results

#### Comparison of experimental results between multi-channel sigma and single-channel sigma

This experiment compares the effects of the Neural Radiance Field (NeRF) based on the multi-channel volume density distribution function proposed in this paper with the original NeRF using a single-channel volume density distribution function in generating images on hyperspectral datasets. The experiment tests the performance of both methods, using a standard NeRF neural network structure with a network width of 256. Training was conducted for 200,000 epochs with early stopping to achieve optimal results. The duration of a single training session was approximately 12 hours on a RTX 2080 graphic card.

Our experiment has shown that, as the dataset size (including approximately uniformly distributed 34-channel hyperspectral datasets with varying image quantities such as 12, 24, 36, 40, 42 images covering 360 degrees) increases, the Peak Signal-to-Noise Ratio (PSNR) value increase, and the proposed method consistently outperforms the results generated by the original NeRF in terms of the PSNR values. The PSNR value curves for both methods across different datasets are depicted in Table 1.

**Table 1:** Comparison of training results between our method (the multi-sigma NeRF) and original hyperspectral NeRF (the single-sigma NeRF) with different training set sizes.

Dataset size	PSNR-single sigma	PSNR-multi sigma	Difference
12	20.31	22.66	+11.5%
24	31.75	32.45	+2.20%
36	32.11	35.54	+10.7%
40	32.53	36.68	+12.7%
42	31.67	37.18	+17.3%

The related SSIM data are shown in Fig. 10 in appendix.

Table 1 illustrates that across all dataset sizes, our method generates hyperspectral images with higher Peak Signal-to-Noise Ratio (PSNR) values compared to the previous method. On average, the PSNR values are approximately 10% higher. For methods like Neural Radiance Fields, increasing the size of the training set generally enables the neural network to obtain more accurate three-dimensional representations, resulting in an increase in the PSNR values of the generated images. As observed in the graph, with an increase in the size of the training set, the generation of hyperspectral images using method proposed by this paper follows this pattern. In contrast, in related experiments with the original hyperspectral NeRF, the PSNR values of generated images reached saturation when the training set size exceeded 24, showing no further improvement. This phenomenon arises due to the following reasons.

For hyperspectral data, many spectral channels introduce inconsistency in three-dimensional information between different perspectives. In the original hyperspectral NeRF method, since the spectral information for all channels is determined by the same volume density distribution function $\sigma$, the convergence of $\sigma$ to an incorrect distribution may occur during training, affecting the pixel rendering results for all channels from all perspectives when noise-induced inconsistencies appear. The erroneous volume density distribution $\sigma$ after rendering results in various noises such as haze, floating spot-like, and spatial distortion, significantly affecting image quality.

When NeRF with a multi-channel volume density distribution function is used for processing hyperspectral data, the issues are alleviated. Addressing the three-dimensional feature inconsistencies in hyperspectral images caused by noise, only some channels fail to obtain a reasonable spatial three-dimensional representation, and their volume density distribution function $\sigma$ is affected. For other channels, $\sigma$ remains unaffected. Moreover, different channels share the implicit space representation vector generated by the same neural network. In the process of error back-propagation, the loss function for most of normal channels carries substantial weight, significantly reducing biases in the spatial representation within the neural network.

**Fig. 1:**
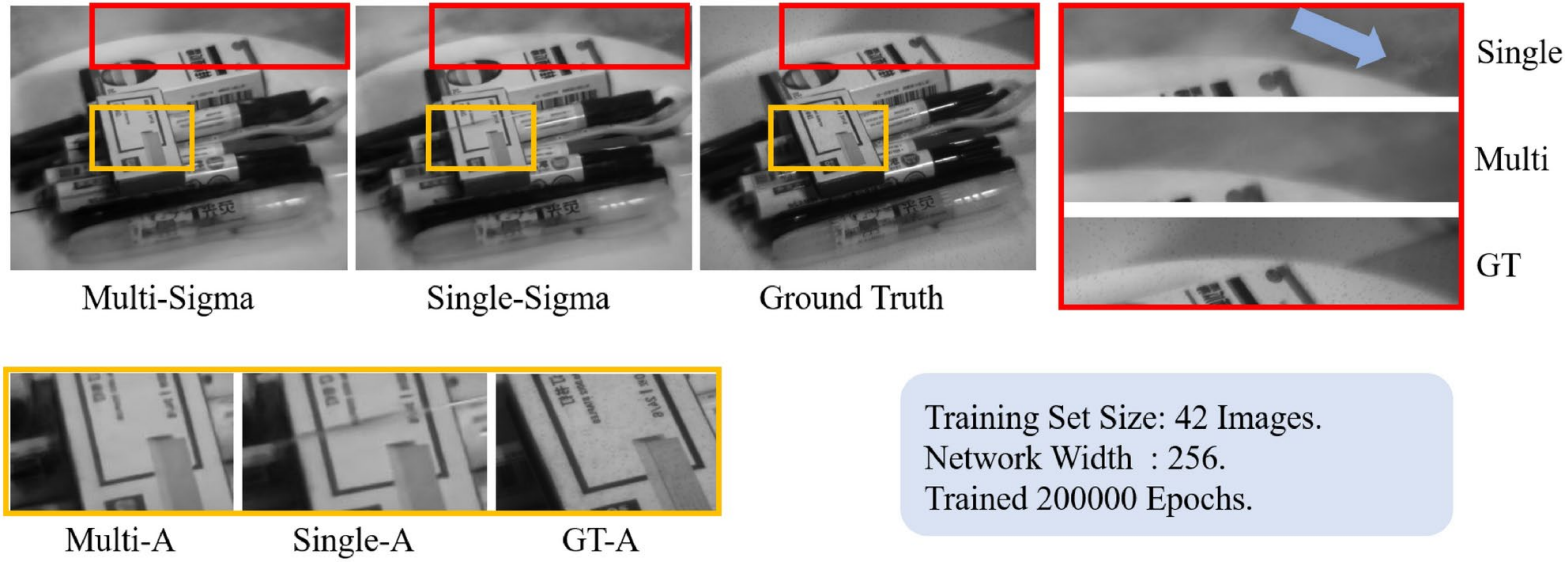
Comparison of output images between both methods and the ground truth. The details in the figure are marked in red and yellow.

Consequently, for three-dimensional feature inconsistencies, each channel’s volume density distribution function only needs to fit three-dimensional features most suitable for that channel based on the space representation vector generated by the neural network with minimal bias. Although this method cannot eliminate noise to achieve a perfect spatial representation, its higher flexibility results in fewer local errors in the rendered images across most channels and perspectives. Some examples are showcased in the Fig. 1.

Figure 1 presents a comparison between the images generated by the method proposed in this paper and the original Neural Radiance Fields (NeRF) method, juxtaposed with ground truth. The spectral image at a wavelength of 600nm is displayed here. The training set utilized 42 images from different perspectives, with a neural network width of 256, and the training extended to 200,000 epochs with early stopping to reach optimal results. Images labeled as “Multi-Sigma” in the graph are generated by our method, while those labeled as “Single-Sigma” are generated by the original NeRF. In the subfigure labeled as “Single-A”, it can be observed that the rendered image exhibits striped noise not present in the ground truth. In contrast, this phenomenon is absent in the proposed method. Additionally, from the images in the red-bordered section on the right, the background rendering in ’Multi’ (ours method) is closer to the ground truth compared to the image ’Single’ (original NeRF method).

**Fig. 2:**
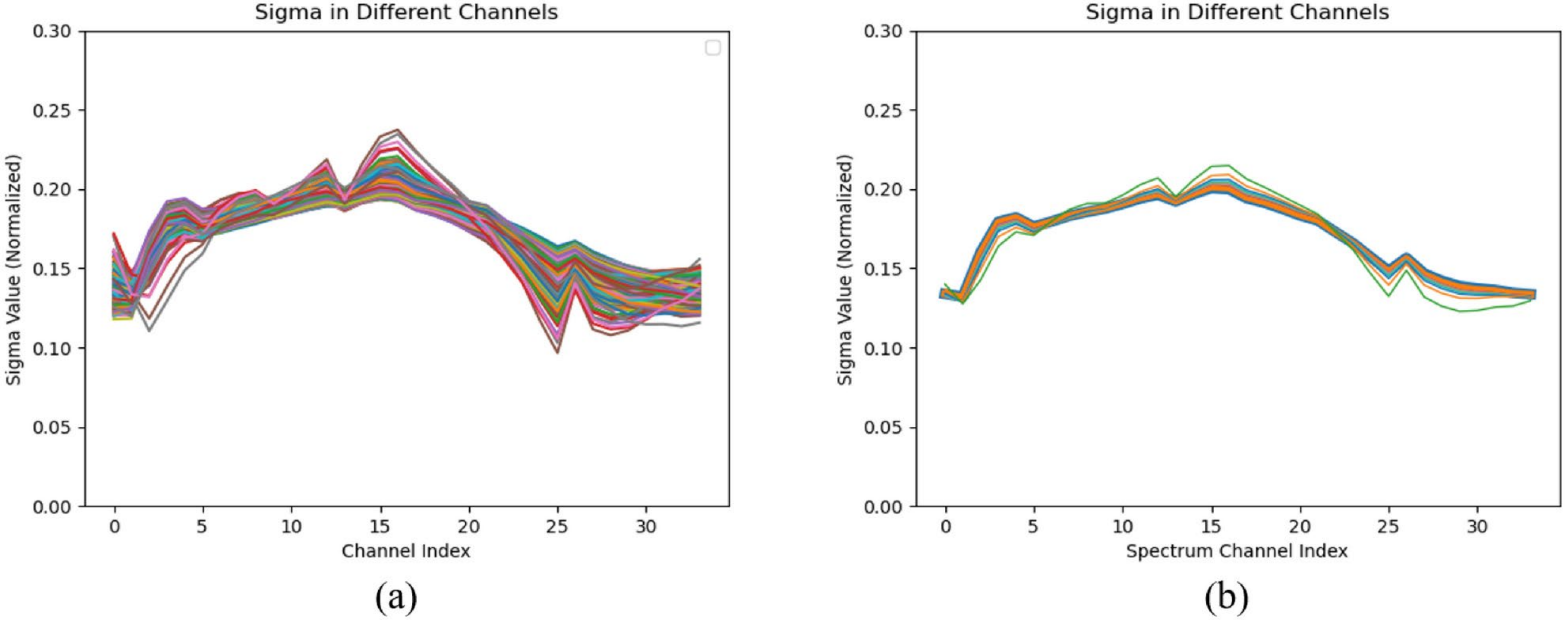
Statistical information of the multi-channel $\sigma$. (**a**) The normalized samples of multi-channel $\sigma$ values as the channel index increases. (**b**) The $\sigma$ curves that appears most frequently in (**a**).

To provide a clearer illustration of the role of the multi-channel volume density distribution function, Fig. 2 presents normalized statistical information of the multi-channel volume density distribution function.

Figure 2 presents the results of overlaying multi-channel $\sigma$ curves for many sampled points, with each $\sigma$ having 34 channels. The results have been normalized because the true $\sigma$ values vary greatly between spatial points, making it inconvenient to display. Figure 2a shows the superimposed curves, while Fig. 2b displays statistically dominant $\sigma$ patterns among the sampled points. Most $\sigma$ curves are close to the mean. Through calculations, it is determined that these curves deviate from the mean by less than 13% on average.

Most curves do not deviate significantly from their own mean value (to some extent the mean value represents the single sigma value in the original NeRF method), but some curves are more prominent. This aligns with the phenomenon observed in the experiments, where the proposed method and the original NeRF method perform similarly well in most regions, but the proposed method optimizes areas where the original NeRF method exhibits errors. The volume density distributions obtained using the multi-channel approach in this paper do not lose their physical meaning. Moreover, they provide higher flexibility for handling noise, inconsistency, or the lack of three-dimensional information in hyperspectral images, leading to better training results. The experiments thoroughly validate the effectiveness of the proposed multi-channel volume density distribution function for rendering hyperspectral images.

#### Investigation on the impact of variation of network width

This section explores the specific role played by the multi-channel volume density distribution function rendering method proposed in this paper. Although the proposed method introduces several thousand to ten thousand parameters to the overall scale of the neural network, the impact on the total parameters of the network is not substantial. However, it is essential to determine whether the improvement in image generation is achieved simply by increasing the number of parameters. In this section, we investigate the differences in the effects produced by the proposed method under different neural network widths (i.e., parameter amounts) to demonstrate the effectiveness and efficiency of our method.

**Fig. 3:**
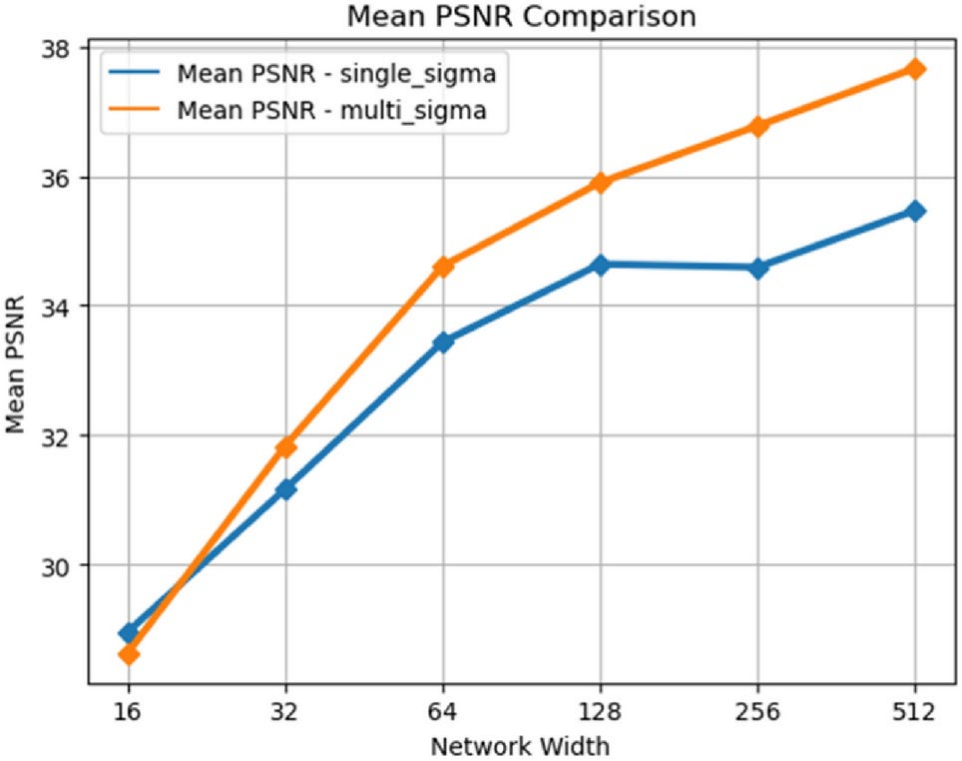
PSNR comparison of images generated by the two methods at different network widths.

Various widths of fully connected layers, ranging from 16 to 512, were used to compose the main structure of the NeRF neural network, while keeping other structures and parameters constant. For each experiment, the network width is doubled compared to the previous experiment, thereby providing a wide range of network experiment results with varying parameter quantities. The experiments involved training for at most 200,000 epochs with early stopping, with 42 images as training set to achieve optimal results. The effects of using the proposed method and the original NeRF were examined for neural networks with different widths. The training duration for a single run ranged from 2 to 17 hours, depending on the size of the network. The training results are illustrated in Fig. 3 Table 2.

**Table 2:** PSNR comparison of images generated by the two methods (the multi-sigma NeRF proposed in this paper and the original single-sigma NeRF) at different network widths.

Network width	PSNR-single sigma	PSNR-multi sigma	Difference
16	28.95	28.62	-1.13%
32	31.16	31.83	+2.15%
64	33.43	34.61	+3.53%
128	34.64	35.90	+3.64%
256	34.59	36.77	+6.30%
512	35.47	37.66	+6.17%

In Fig. 3 and Table 2, the PSNR values of images rendered by NeRF using the multi-channel volume density distribution function method are superior to the original NeRF for almost all neural network widths. In most cases, the advantage exceeds 3.5%. Additionally, both PSNR curves exhibit saturation when the width exceeds 128, indicating that further increases in width do not significantly improve PSNR values. In contrast, the NeRF output PSNR using the proposed method is noticeably higher than the original PSNR. This suggests that the effectiveness of the proposed method is not due to a simple increase in the number of parameters.

From another perspective, the additional parameters introduced by our method are only the product of the channel count and the network width. The increase in the number of parameters is much smaller than the increase in parameters resulting from widening the fully connected layer. However, the improvement in PSNR values obtained by this method is comparable to, and even higher than, the improvement in PSNR values resulting from an increase in network width. This strongly demonstrates the high parameter efficiency of the proposed method in the hyperspectral domain and achieves significant improvement with a minimal increase in parameters.

The primary factor contributing to this phenomenon is that the additional parameters introduced by our method and the parameters increased by widening the fully connected layer have different roles in the neural network. As observed in images and referenced from previous studies, increasing the width mainly enhances the expressive capacity of the neural network. Wider neural networks output images with more details, and the spatial structure of the main objects is more accurate. The main function of the proposed method is to enhance the flexibility of volume density distribution functions in different channels by partially decoupling them. This, in turn, improves the accuracy of NeRF in handling multi-channel hyperspectral datasets, especially in the presence of noise and inconsistency.

When the network width is 16, the representation capacity of the neural network is very low due to lack of parameters. These networks have poor performance with low PSNR and SSIM, indicating they are not learning correct representations of objects. Our method does not enhance the representation capacity of the neural network since only few parameters are added compared with the original method. Thus, our method shows no advantage when the network width is 16 or lower and it is not a recommended hyper-parameter for our method.

**Fig. 4:**
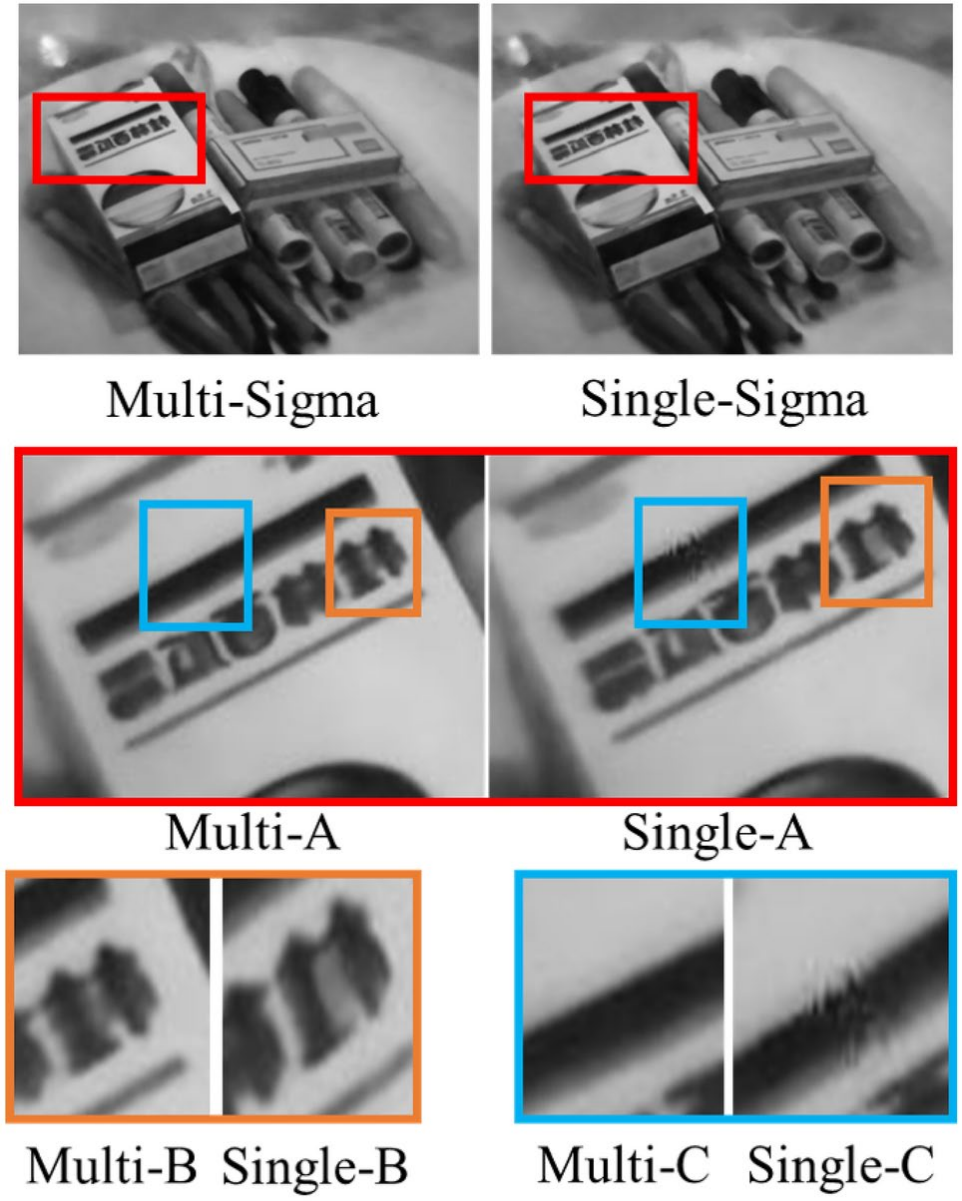
Comparison of output images between the proposed method and the original hyperspectral NeRF. There are more small fuzzy areas caused by small floating objects in single channel method than those in our method.

To show the difference between the results of two methods, Fig. 4 shows the generated images.

**Fig. 5:**
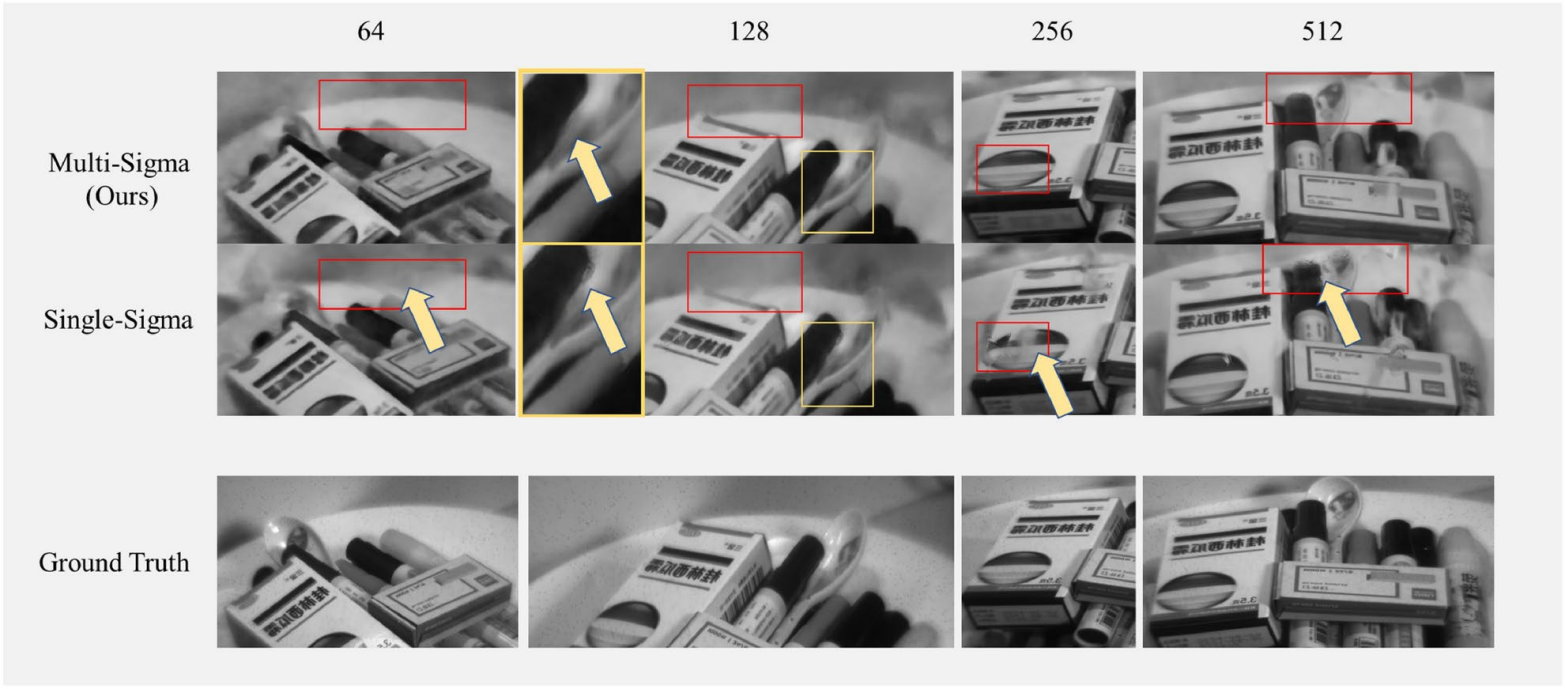
Comparison of output image quality between the two methods with different network widths. The top row contains images generated by our method, the middle row contains images generated by original hyperspectral NeRF. Each column in the above rows stands for the output of specific network width. The bottom row contains the ground truth images. There are more fuzzy areas caused by floating objects in images generated by single sigma method than those by ours. One example is the small floating object ahead of the yellow arrow in the left-bottom zoomed in image with yellow rectangles.

Examples in Fig. 4 were trained in both methods under network width 128, images of 600nm wavelength are displayed. The method proposed in this paper slightly enhances the accuracy of detail expression (Multi-B, Single-B) and corrects errors in some training results of the original NeRF method (Single-C). Even when the neural network adopts our method, a certain width of neural network fully connected layers remains essential. Although, in terms of PSNR values shown in Fig. 3, the proposed method can generate images with quality comparable to higher network widths in the original NeRF, visually, the proposed method effectively enhances the accuracy of images in inconsistent regions. However, there is no significant improvement in representational capacity, lacking sufficient detail. This also demonstrates that our method does not achieve better results by simply increasing the number of parameters. To illustrate the differences between the two methods at different network widths more clearly, Fig. 5 is presented.

The spectral image at a wavelength of 600nm is displayed in Fig. 5, which presents the results of training the proposed method and the original NeRF at various network widths, with the first row showing the outcomes of the proposed method and the second row displaying those of the original NeRF. In the first row, which represents the proposed method, the method performs well in correcting errors present in the original NeRF across different network widths.

The experimental results serve as robust evidence that the proposed method does not rely on increasing the number of parameters to enhance the quality of the output images. While the proposed method significantly improves image quality and corrects errors, it cannot replace the need for a neural network with a certain parameter scale to adequately support and achieve optimal results. This highlights the nuanced role of the proposed method in enhancing the performance of neural networks.

#### Investigating the impact of different spectral channel numbers on single and multi-channel sigma

This section investigates whether the method proposed in this paper remains effective for data like RGB. The proposed method is tailored for hyperspectral data characterized by high volume and noise. Its effectiveness might decrease on datasets like RGB, which lack these specific features. We conducted experiments on images with different channel numbers. In this experiment, we selected different channel quantities using a uniform interval method, ensuring minimal influence from brightness factors between channels.

**Fig. 6:**
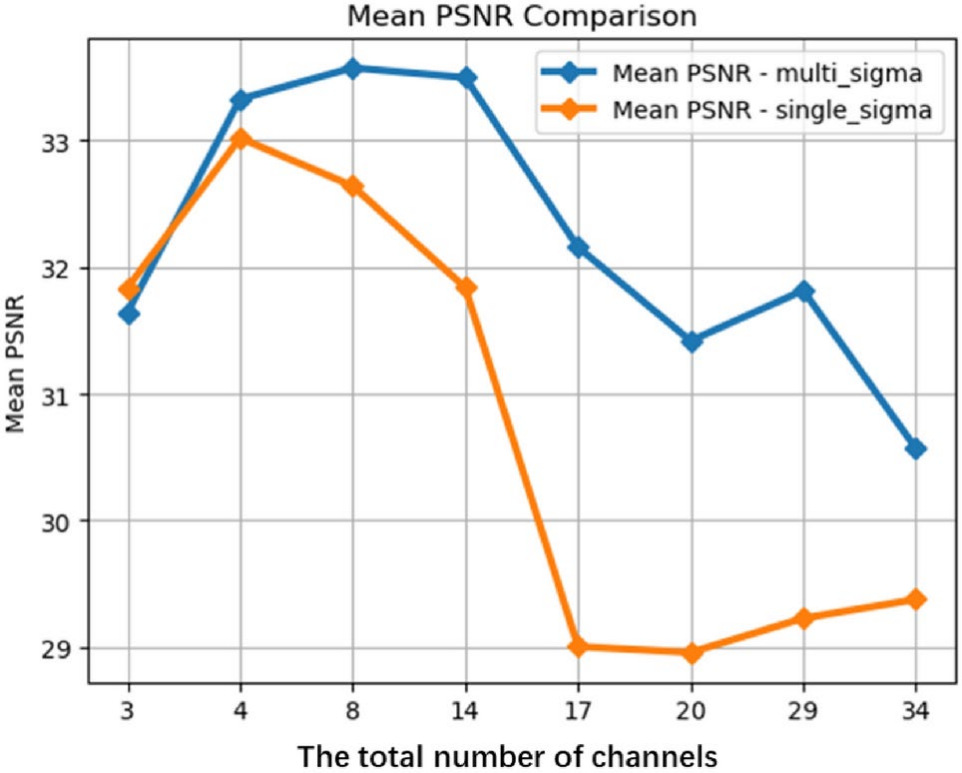
Performance comparison of output images between the two methods trained on datasets with different channel numbers.

For each training set variation, we maintained consistent neural network parameters. Specifically, a standard NeRF structure with a width of 256. The training was conducted for 50,000 epochs, during which overfitting was not observed. The experiment tested the impact of using the proposed multi-channel volume density distribution function rendering method on the results, illustrated by the two curves in Fig. 6 and details in Table 3.

**Table 3:** Performance comparison of output images between the two methods (the multi-sigma NeRF proposed in this paper and the original single-sigma NeRF) trained on datasets with different channel numbers.

Channel num	PSNR-single sigma	PSNR-multi sigma	Difference
3	31.83	31.64	-0.5%
4	33.02	33.32	+0.9%
8	32.64	33.57	+2.8%
14	31.83	33.49	+5.2%
17	29.00	32.15	+10.8%
20	28.95	31.41	+8.8%
29	29.22	31.81	+8.4%
34	29.37	30.56	+4.4%

Figure 6 depicts the curves of PSNR values for both methods as the number of training set image channels varies. Table 3 displays the details. From Fig. 6, when the number of channels is relatively low, the proposed method in this paper does not exhibit significant improvement. However, as the number of channels increases, the original NeRF method shows a noticeable decline in performance on such datasets, with a decrease in PSNR values by approximately 4. In contrast, when using the proposed method for rendering, the PSNR values only decrease by 2. As the number of channels increases, the results of training with the proposed method outperform those of the original NeRF method, with PSNR values surpassing by 2.8% to 10.8%. This further substantiates the effectiveness of our method in the hyperspectral domain.

The output image PSNR does not significantly improve when using the proposed method with an image of only few channels. This phenomenon is attributed to the limited three-dimensional representation information provided by the lower volume of image data with few channels compared to hyperspectral images. Increasing the parameter scale or flexibility using the proposed method does not lead to further improvement in this scenario.

With the increase in the number of channels, the PSNR of the rendered images by the neural network continuously decreases. This phenomenon arises due to the substantial increase in input data volume, requiring a proportional increase in the neural network’s representational capacity to achieve similar results. Furthermore, as the inconsistency and noise in multi-channel images also increase, maintaining the same neural network scale leads to a natural decline in training results. When the network scale increases, the PSNR of the neural network’s output results gradually improves.

In summary, our method is optimized for the characteristics of hyperspectral data, showing significant effectiveness when the spectral channel count is high. However, in domains with lower channel counts, such as RGB images, our method may not fully exert its benefits.

## Discussion

This paper addresses challenges in NeRF-based 3D reconstruction for hyperspectral images and introduces a method to improve NeRF’s effectiveness in handling such data. Hyperspectral datasets are characterized by large volumes of data, significant noise, and inconsistencies, leading to convergence errors during NeRF reconstruction. To tackle these issues, we propose a new NeRF rendering method specifically designed for hyperspectral datasets, utilizing a multi-channel volume density distribution function. Numerous experiments demonstrate the clear optimization effect of this method for hyperspectral datasets, achieving substantial improvements in PSNR with only a modest increase in parameters.

Our approach targets challenges such as noise and inconsistency in hyperspectral datasets by employing a multi-channel volume density distribution function that shares the same NeRF latent space representation vector. The inherent noise and inconsistency of the hyperspectral data cause a lot of problems in novel view synthesis, and one of the most important one is generating erroneous floating objects. The floating objects are generated by noise and inconsistency of hyperspectral images. The incorrect positions and shapes of the floating objects lead to local distortions and low resolution in the rendered images of multiple different perspectives. This problem is more severe in hyperspectral data because any inconsistency or noise in any channel can generate floating objects, and the impact spread to all channels through shared opacity. Hyperspectral data have far more channels then RGB images, and thus the probability of floating objects appearing increases significantly. To address this problem, our method adapts channel-wise opacity instead of shared opacity, offering more flexibility for opacity of a given position. At a certain position, even if a particular channel encounters inconsistency and produces floating objects, the other channels are not affected by this channel, maintaining the reliability of the generated results in other channels.

This approach partially decouples the three-dimensional representations between different channels, enhancing NeRF’s spectral flexibility. Consequently, it better renders inconsistent local regions in images, reducing the impact of erroneous convergence on other perspectives and enhancing overall imaging quality. The substantial data volume in hyperspectral datasets provides ample spatial information constraints, ensuring the correct convergence of hidden spatial representation vectors. Even with the decoupling of volume density distribution functions among different channels, this does not lead to significant divergence in spatial distribution between channels, ensuring accurate convergence for each channel. Thus, our proposed method not only improves imaging quality in hyperspectral datasets but also ensures correct convergence for the three-dimensional representations of each channel, reflected in increased accuracy in local image regions and mitigated impact from erroneous convergence on other perspectives. Our experiments indicate that our method does not enhance the neural network’s ability to represent details; the richness of image details depends on the width of the neural network’s main fully connected layer. Therefore, our method achieves optimal results when used in conjunction with a neural network of sufficient width.

Experiments on datasets with low channel counts show that our method does not yield significant improvements. As these datasets lack characteristics such as high data volume, substantial noise, and inconsistent regions, our method does not contribute to the generation of higher-quality images. This conclusion aligns with our theoretical analysis. Overall, our method effectively enhances NeRF’s reconstruction performance for hyperspectral image datasets under reasonable neural network parameter configurations, with potential extensions for tasks like hyperspectral dataset augmentation.

There are areas that need to be improved in the future. Our method conducted neural networks to represent the 3D information, which is relatively slow in rendering and no explicit surface can be represented. In the future research, faster methods and methods that can represent explicit surfaces can be explored.

## Conclusion

This paper introduces a rendering method for Neural Radiance Fields (NeRF) tailored to the unique characteristics of hyperspectral datasets. Through a series of experiments, we demonstrate the efficacy of this method in enhancing the quality of generated hyperspectral images while minimizing the impact of convergence errors across multiple perspectives. In summary, our proposed approach, when combined with appropriate neural network parameters, proves instrumental in elevating NeRF’s generation performance specifically for hyperspectral images.

## Methods

**Fig. 7:**
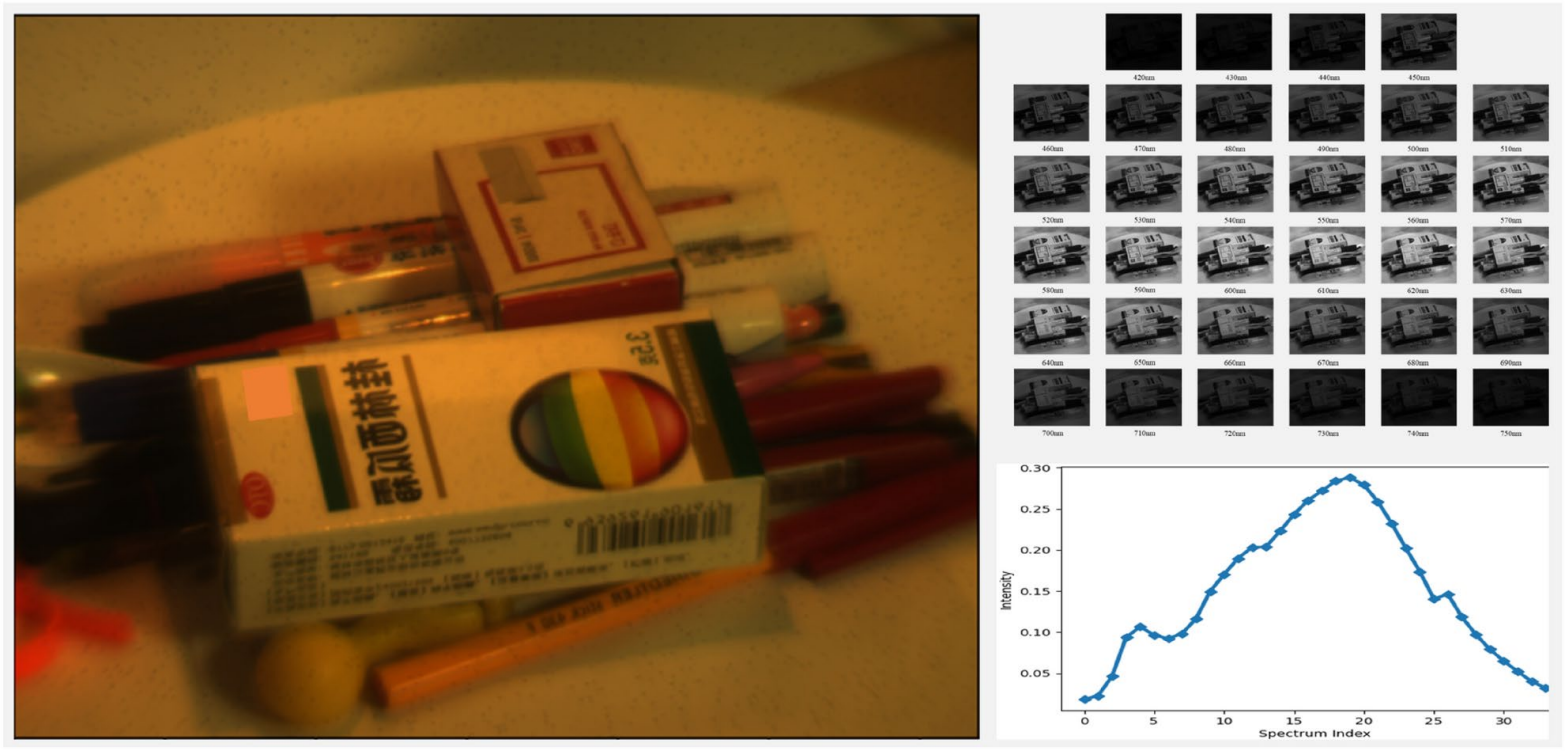
An example of hyperspectral images. The image on the left is the RGB image recovered from a hyperspectral image. The images on the right top show each channel of a hyperspectral image. The image on the right bottom illustrates a canonical spectrum of the hyperspectral image. The spectrum index corresponds with spectrum ranging from 420nm to 750nm, with a spectral gap of 10nm between each channel. The size of each image is 640 by 480.

This section delves into the specifics of the method introduced in this paper. Initially, we will discuss certain shared details related to NeRF, followed by a comprehensive presentation of our methodology. A brief introduction to hyperspectral images we used is illustrated in Fig. 7.

### Neural radiance fields

NeRF is a neural network-based technique for synthesizing new perspectives. This method trains a neural network using images from various angles to implicitly represent the three-dimensional distribution density of the target object and intensity information from different channels. Subsequently, based on this three-dimensional distribution information, the method samples rays at corresponding angles and renders images from any perspective using a camera model. A brief introduction of NeRF are illustrated below, further detatils and flow charts can be found in NeRF^12^.

#### Neural rendering

 In the original Neural Radiance Fields (NeRF) method, the neural network takes as input spatially encoded three-dimensional coordinates and two angular directions (pitch and yaw), totaling five dimensions. It outputs the value of the three-dimensional volume density distribution function at that point, denoted as $\sigma (x, y, z, \gamma , \phi )$, and the color information at that point and viewpoint, represented by the vector $\vec {c}$. During rendering, the final pixel color value is obtained by calculating the aforementioned information for all sampled points along the path.

The neural network core of NeRF consists of 8 fully connected layers, along with corresponding output layers for density distribution and intensity information in different channels. The input to the neural network is the coordinates of sampled points, encoded with positional information, and directional details. After passing through the neural network, calculations yield volume density distribution and color information for each coordinate. During the generation of an image from a specific viewpoint, pixel-wise rendering is necessary. The value of each pixel is determined by sampling the density distribution and color at a series of coordinates along the ray determined by the camera model.

#### Position encoding

 NeRF doesn’t directly input three-dimensional coordinates into the neural network. Instead, it uses position encoding:

1 $$\begin{aligned} \gamma (p) = (sin(2^0 \pi p), cos(2^0 \pi p), ..., sin(2^{L-1}\pi p), cos(2^{L-1}\pi p)) \end{aligned}$$

P is the three-dimensional coordinate of a given point. This is because neural networks tend to prioritize fitting low-frequency data and may not fit high-frequency data well. Position encoding improves the network’s ability to represent high-frequency data effectively, thereby enhancing the training of the neural network. Position encoding is also adapted into our experiment, the value of L for the position encoding used during training is 10.

#### Training

 During the training of the neural network, pixels of the training sample images are sampled. The coordinates along the rays generated by the camera model are then fed into the neural network. The network outputs density distribution and color information for the corresponding coordinates. Subsequently, the neural network’s output for that pixel is obtained. The loss function is calculated based on the difference between this output and the true values of the samples. The error backpropagation algorithm is employed to update the network’s parameters, facilitating training.

#### Issues

 Theoretically, NeRF have reasonable physical assumptions and strong expressive power. In practice, in the RGB domain, NeRF performs well when the three-dimensional information from various angle images is consistent, and there are a sufficient number of viewpoints. However, training NeRF neural networks with multiple viewpoints containing inconsistent information widely leads to convergence issues, especially when the number of viewpoints is limited. Inconsistent multi-view images make it challenging for the neural network to accurately establish the three-dimensional spatial distribution, requiring the network to output color information that varies abruptly with the viewpoint. The neural network exhibits high noise and struggles with handling abrupt features during these processes.

In the hyperspectral NeRF domain, the mentioned issues become more severe due to the characteristics of hyperspectral datasets, including multiple channels, high data volume, high noise, and potential inconsistencies. This necessitates more viewpoints and higher-quality captures for hyperspectral datasets, but obtaining such datasets is inherently challenging. Different hyperspectral cameras have varying acquisition speeds and pixel resolutions, and noise problems in hyperspectral datasets are hard to match with the level seen in RGB cameras. Under past conditions, addressing these issues involved using larger-scale neural networks to enhance expressive power, but experimental outcomes showed minimal effectiveness. The above issues lead to our approach.

### Neural radiance fields rendering based on multi-channel volume density distribution function

In this section, details of the method employed in this paper will be explained. The original Neural Radiance Fields (NeRF) method renders all channel information using the same volume density distribution function. This approach yields suboptimal results when applied to hyperspectral datasets, leading to issues such as erroneous convergence and degraded rendering quality. These problems significantly impact the quality of the generated images. The proposed method in this paper addresses these issues by splitting the volume density distribution function into multiple channels. Each channel is responsible for rendering the spectral information for its corresponding aspect. This approach effectively resolves the previously mentioned problems, enhancing the rendering performance and image quality, especially when dealing with complex datasets like hyperspectral datasets.

**Fig. 8:**
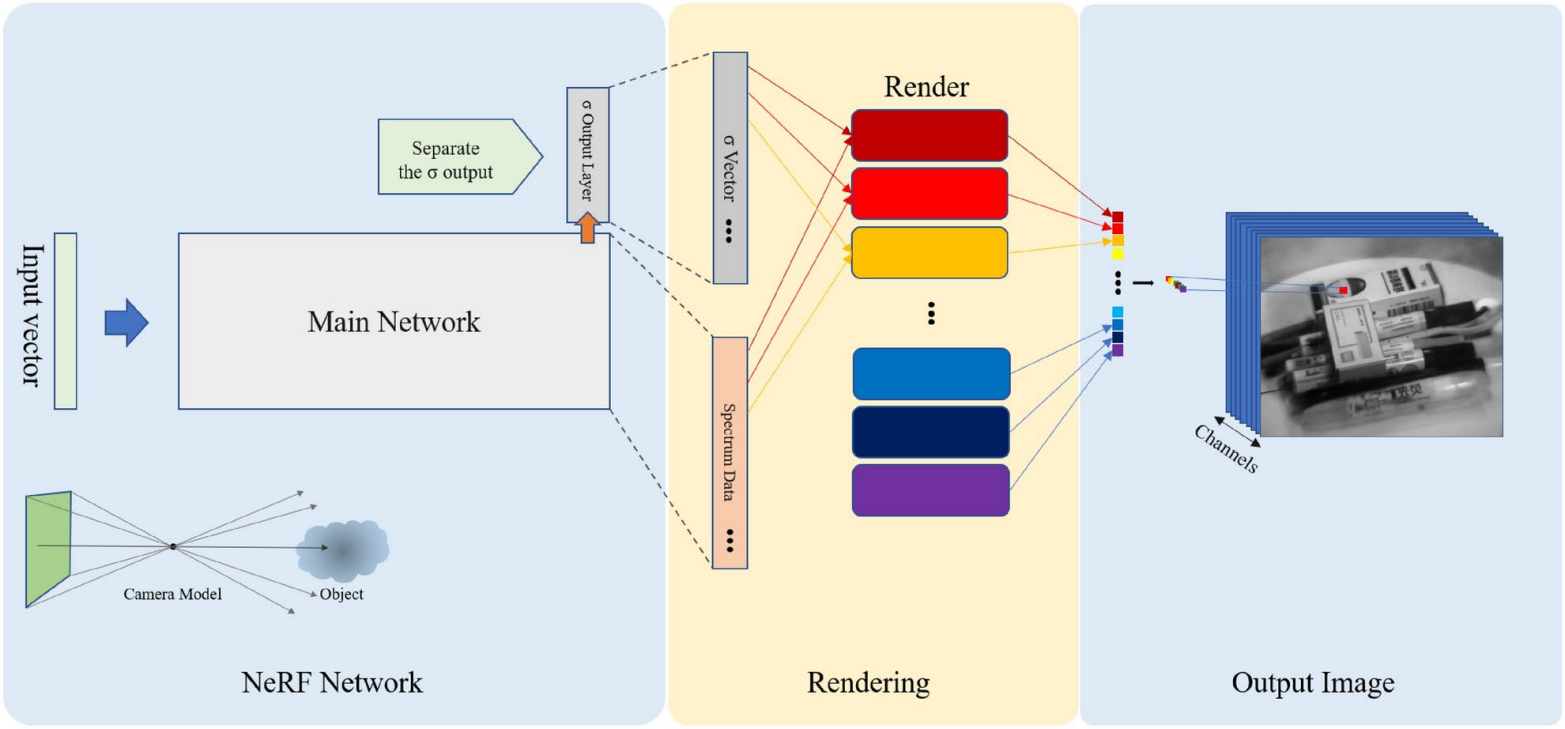
Multi-channel volume density distribution rendering method for hyperspectral imaging in neural radiance fields.

The schematic representation of the proposed method is depicted in the Fig. 8. The input vector to the neural network consists of the sampled positions and viewpoint information based on the lines of sight generated by the camera model. After inputting this information into the main network, two components are produced. The upper part represents the volume density distribution function $\sigma$, while the lower part corresponds to the spectral information from different channels.

In the method, the $\sigma$ value transitions from a scalar to a vector. This vector has dimensions consistent with the number of hyperspectral channels, where each dimension’s volume density distribution value only influences the rendering of the hyperspectral data for that specific channel. In hyperspectral datasets, there are not only the three RGB channels but potentially dozens of different channels. Each pixel can be represented as a vector, considering the volume density distribution values for each channel:

2 $$\begin{aligned} \hat{C}(r) = [\hat{C}_{channel=1}(r), \hat{C}_{channel=2}(r), ..., \hat{C}_{channel=n}(r)] \end{aligned}$$

$\hat{C}(r)$ represents the color information for the pixel corresponding to the r ray, which is a three-dimensional vector in RGB images or a vector with dimensions equal to the number of hyperspectral channels, such as 34 in this study. Each channel of the pixel corresponds to a dimension of the vector. Consequently, in the method proposed in this paper, the rendering process for each channel is defined by the following formula:

3 $$\begin{aligned} \hat{C}_{channel}(r)= & \sum _{i=1}^{N}{T_i}_{channel}(1-exp(-{\sigma _i}_{channel} \delta _i)){c_i}_{channel} \end{aligned}$$

4 $$\begin{aligned} {T_i}_{channel}= & exp\left( -\sum _{j=1}^{i-1}{\sigma _j}_{channel}\delta _j\right) \end{aligned}$$

$\hat{C}_{channel}(r)$ represents the intensity value of the pixel generated in the current channel, which is a scalar. ${\sigma _i}_{channel}$ indicates the volume density distribution function for the current channel at position i. ${c_i}_{channel}$ signifies the intensity value of the current channel at position i. ${T_i}_{channel}$ signifies the probability that the ray starting from the camera center can reach this point without obstruction at the i-th sampled point for the current spectrum channel. $\delta _i$ denotes the distance interval sampled at point i, which remains the same in different channels. The formula indicates that each pixel from each channel is rendered from the volume density distribution and color information of the sampled points along its corresponding ray path. To generate the entire image, each pixel needs to be rendered sequentially.

The proposed method implies that the volume density distribution for different spectral channels doesn’t need to remain consistent. Instead, it can be optimized separately for different channels based on image information. We conducted experiments from various perspectives to validate this method, demonstrating its superior performance compared to the original NeRF under various conditions. The improvement in performance is not solely attributed to an increase in the number of parameters.

**Fig. 9:**
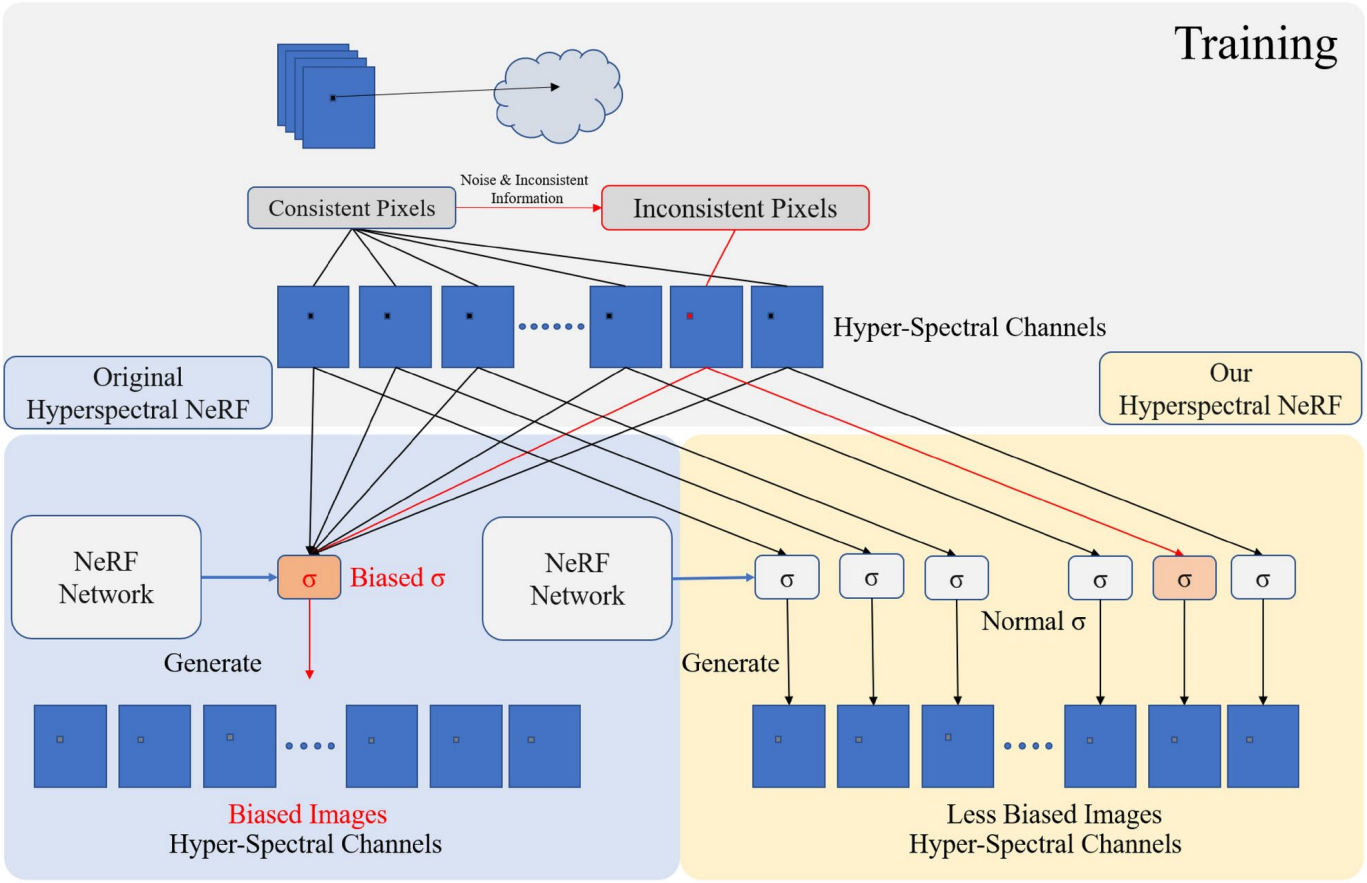
Comparison between multi-channel volume density distribution method and the original method. The upper part is the common training procedure, where the left bottom part shows the original NeRF method and the right bottom part illustrates the method proposed in this paper. On the top of the figure, the cloud signifies a specific object.

**Fig. 10:**
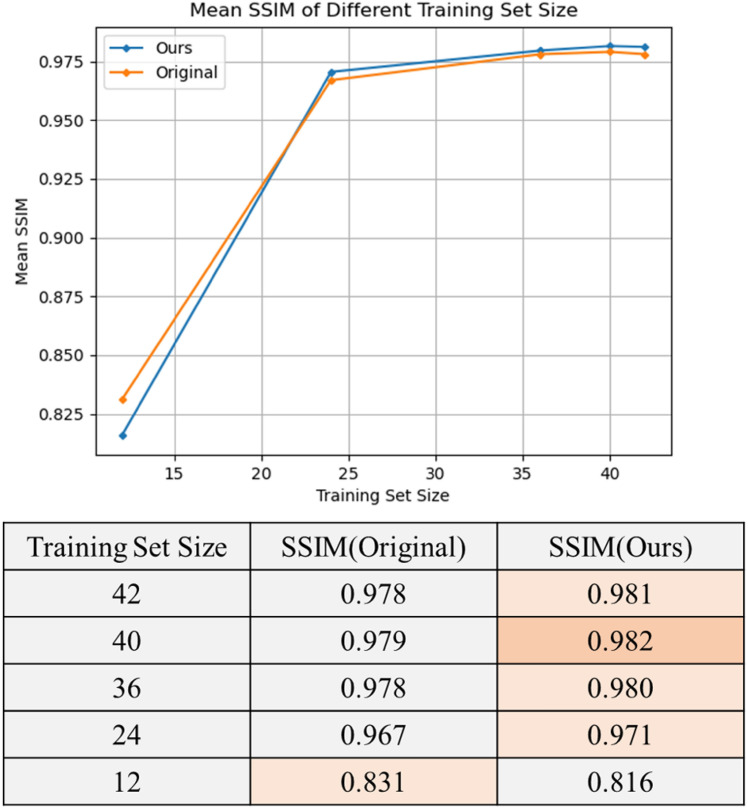
SSIM data related with PSNR data in Fig. 1. The SSIM values of our method are higher then the SSIM of the original method.

To provide a detailed comparison of the rendering and training approaches for hyperspectral datasets, we contrast the method proposed in this paper with the original NeRF method in Fig. 9.

The upper part of Fig. 9 reveals that in hyperspectral images composed of multiple channels, some channel pixels deviate from their correct values due to the influence of noise. If the deviation is caused by inconsistent perspectives, all channels at that point will exhibit deviation. When the image undergoes neural network training, it results in varying degrees of distortion in the spatial distribution function. In the original NeRF method, this phenomenon causes the neural network to converge to incorrect spatial distribution functions, leading to sampling higher density distributions in spaces where there should be no objects, causing significant noise interference in the image.

With the adoption of our method, when certain channels deviate, only specific channels are influenced by noise. Since the spatial distribution functions for all channels are calculated from the implicit spatial distribution vector of the NeRF neural network, it provides relatively accurate spatial information cues for these channels. Consequently, the convergence results are better than the original NeRF’s volume density distribution function, resulting in images that are closer to the original.

Our method also allows each channel to find its local optimal solution, improving the accuracy of generated images. The original NeRF structure, in such scenarios, is compelled to render incorrect objects, leading to poorer image quality. In cases of image inconsistency, our method cannot guarantee that the NeRF neural network obtains a more accurate implicit spatial representation during training. This is because the image information lacks precise and consistent three-dimensional spatial cues, and neural networks cannot create information out of nothing. However, our method can, in such situations, generate images closer to the true values through local optimal solutions^46^.

## Data Availability

The datasets generated and/or analyzed during the current study are not publicly available due to the authors of this article do not have full rights to this dataset, but are available from the corresponding author on reasonable request.

## Appendix

See Fig. 10.

## Code availability

The code of this paper is available at https://doi.org/10.5281/zenodo.15073781.
